# Computer-Aided Drug Design of Natural Candidates for the Treatment of Non-Communicable Diseases

**DOI:** 10.1155/2022/9769173

**Published:** 2022-06-26

**Authors:** Hilal Zaid, Siba Shanak, Akhilesh K. Tamrakar

**Affiliations:** ^1^Qasemi Research Center-Al-Qasemi Academy, P.O. Box 124, Baqa El-Gharbia 30100, Israel; ^2^Faculty of Medicine, Arab American University, P.O. Box 240, Jenin, State of Palestine; ^3^Faculty of Sciences, Arab American University, P.O. Box 240, Jenin, State of Palestine; ^4^CSIR-Central Drug Research Institute, Lucknow 226031, India

Noncommunicable diseases (NCDs) impose a significant burden on the health care systems in developed and developing countries. Indeed, the incidence of NCDs (e.g., diabetes, cancers, chronic respiratory diseases, and cardiovascular diseases) has increased in epidemic proportions worldwide [1]. According to estimates made by the WHO, about 41 million people die annually worldwide, equivalent to 71% of all deaths, because of NCDs. 37% of those who died with NCDs are between the ages 30 and 69 years old. Lack of access to essential medicines for NCDs is a major challenge, especially in developing countries [2, 3].

Drug discovery for medicinal plant provides important leads against various pharmacological targets. A large number of plants used in the traditional medicine have now become a part of the modern world health care system. Natural novel drugs are now more achievable due to modern techniques for separation, structure elucidation, screening, and bio- and chemo-informatics [4, 5].

Over the last decades, the amount of biological and chemical information has escalated drastically. Thus, the need for new scientific branches that handle big data has arisen. Computational chemistry and bioinformatics are now well-established scientific fields that offer the scientific community the opportunity to study drug-disease relationship. One such way to study this relationship is via understanding the connection of the disease to the target, and the consequent mechanism of interaction between the target and the drug [6, 7].

The heterogeneity and lacking of enough data can be an obstacle that hinders the mechanistic comprehension of drug-disease relationship. In this regard, predictive computational approaches in the field of computational chemistry, e.g., network pharmacology approaches, predictive ADMET (absorption, distribution, metabolism, and elimination-toxicity), and pharmacophore modeling, can be used to build scientific algorithms, which are developed to overcome this barrier [8, 9]. Such approaches are cost-effective in identifying drug candidates, as they limit the extensive use of animal models against all plausible lead compounds, while identifying safe and effective drugs. Within this field of research, we launched a research topic entitled “Computer-Aided Drug Design of Natural Candidates for the Treatment of Non-Communicable Diseases.” For the aforementioned reasons, this research topic attracted the attention of scientists and received a number of submitted manuscripts. Twenty-one papers were submitted for publication in this issue. After an extensive review process, thirteen original research articles have been published within this special research topic. These research articles cover various topics in drug design, reporting advance in *in silico* methods in drug discovery.

Network pharmacology has emerged as an important tool to understand the mechanism of action of a drug/drug candidate. It uses computational power to systematically catalogue the molecular interactions of a drug molecule in a living cell. Hu et al. exploited network pharmacology and data mining to elucidate the role of traditional Chinese medicine (TCM) in relieving epidermal growth factor receptor-tyrosine kinase inhibitor (EGFR–TKI)-associated diarrhea. Using various approaches, they obtained 23 potential therapeutic TCM targets for the treatment of EGFR-TKI-related diarrhea. Based on the findings, they proposed that TCMs can provide data to support experimental and clinical studies on the relief of EGFR-TKI-associated diarrhea. Concomitantly, Xu et al. presented a pioneer report on the use of TCM to treat summer fever, especially in children.

Yun et al. aimed to provide the basis for understanding the mechanisms of action for active ingredients of TCM against angiogenesis. GeneCards was used to explore angiogenesis-related targets. The TCM system pharmacology platform was used to uncover natural compounds. Target-compound, compound-medicine, and target-compound-medicine networks were constructed using Cytoscape. To predict the target-compound binding, molecular docking was used. Of 79 targets for angiogenesis, 41 targets were matched to 3839 compounds. 110 compounds from the dataset were found to have high correlation with angiogenesis. As a result of molecular docking, fifty-five combinations were constructed. Top combinations were for PTGS2-astragalin (−9.18 kcal/mol), KDR-astragalin (−7.94 kcal/mol), PTGS2-quercetin (−7.41 kcal/mol), and PTGS2-myricetin (−7.21 kcal/mol). Using the network, eighty other combinations were obtained and classified according to their affinities. Top combinations were KDR-beta-carotene (−10.13 kcal/mol), MMP9-beta-sitosterol (−8.04 kcal/mol), MMP9-astragalin (−7.82 kcal/mol), and MMP9-diosgenin (−7.51 kcal/mol).

Cheng et al. described use of network pharmacology to investigate the mechanism of action of allicin to modulate lipid metabolism for the management of NAFLD. Using an *in vitro* cellular model, they predicted two hundred and nineteen potential targets of allicin PharmMapper. Out of those, 44 potential targets related to lipid metabolism were screened out as protein targets of allicin. In the same line, Ni et al. determined pharmacological mechanisms of Chinese incompatible herbs Fuzi and Banxia (FB) in chronic obstructive pulmonary disease (COPD) using network pharmacology. From the identified targets, they proposed that effect of FB against COPD may involve the regulation of immunological function. This study provides an excellent example of the application of network pharmacology in evaluating mechanisms of action and molecular targets of an herb. Another study by Dan et al. explored the material basis and the rule of TCM against antineoplastic drug-induced cardiotoxicity (ADIC) using network pharmacology and data mining. They identified 21 potential targets, 332 candidate compounds, and 400 kinds of herbs for the management of ADIC.

Two new tyrosinase inhibitors from the stems of *Streblus ilicifolius* were reported by Nguyen et al. One molecule possesses strong tyrosinase inhibitory activity with an IC_50_ value of 0.01 *µ*M. Docking studies showed different binding affinity of the two molecules for *oxy*-tyrosinase. An independent study by Bader et al. presented the design and synthesis of 4-O-podophyllotoxin sulfamate derivatives and their evaluation in various cancer cell lines as potential cytotoxic agents.

Yu et al. conducted network pharmacology to uncover the possible mechanisms in Fuxin mixture or FXHJ for the treatment of heart failure. Ingredients of FXHJ were analysed, and 39 active ingredients were explored. 47 action targets were found to bind to these compounds. The network was constructed and enrichment analysis undertaken. The treatment of heart failure by FXHJ mixture was predicted to be via regulating several cascades, including the MAPK signaling pathway, PI3K/Akt signaling pathway, cAMP signaling pathway, TNF signaling pathway, toll-like receptor signaling pathway, VEGF signaling pathway, NF-kappa B signaling pathway, and the apoptotic signaling molecule BCL2.

The manuscript by Shanak et al. presented the inhibitory effect of the *Ocimum basilicum* extract and its major constituents on *α*-amylase and *α*-glucosidase using *in vitro* and *in silico* techniques and proposed *Ocimum basilicum* as a potential source to identify antidiabetic leads [10]. Similarly, Dang et al. conduced an in vitro and in silico study, where two new diarylalkanoids, semedienone, and semetrienone were isolated from a CHCl_3_-soluble extract of the stems of *Semecarpus caudata* (Anacardiaceae), and their structures were resolved using NMR. These compounds were analysed for their inhibitory activity against tyrosinase inhibitory activity, and the IC_50_ values were 0.033 and 0.11 *μ*M, respectively. In silico docking studies of the two compounds against *oxy*-tyrosinase were carried out to analyze their interactions. Of the two compounds, semedienone showed decent interactions with the amino acid and peroxide group residues of the target enzyme.

Protein alpha synuclein is a protein that shows high accumulation during Parkinson's disease. Prolyl oligopeptidase (POP) is a serine protease that was shown to affect the accumulation of alpha synuclein and is a key target for the treatment of Parkinson's disease. Kulkarni et al. carried out an *in silico* study to evaluate the efficacy of an alkaloid class of phytochemicals against POP. Chemical Entities of Biological Interest (ChEBI), a publicly available database was used to retrieve the chemicals. Discovery Studio was used to predict the ADMET properties of the alkaloids to calculate their drug likeness using the Lipinski's rule of 5 and to filter the parameters based on their feasibility for the central nervous system and to cross the blood-brain barrier (BBB). To scan the strength of compound-target binding, molecular docking was performed followed by molecular dynamic (MD) simulations that enable checking the stability of alkaloid-protein complexes. The following alkaloids were selected as plausible lead compounds against POP: metergoline, pipercallosine, celacinnine, lobeline, cystodytin G, lycoperine A, hookerianamide J and martefragin A. Among these, metergoline, pipercallosine, hookerianamide J, and lobeline showed the most promising results comparable docking scores to three POP inhibitors that had reached clinical trials, i.e., Z-321, S-17092, and JTP-4819. MD simulations showed high stability of the plausible lead compounds in the active site when compared to the binding modes of the known inhibitors.

Myelin and lymphocyte and T cell differentiation protein 2 (MAL2) are expressed in several forms of cancer, including breast cancer. Zhong et al. studied the relationship between the expression of MAL2 and breast cancer using the Oncomine database and the Cancer Genome Atlas database. Quantitative real-time polymerase chain reaction (RT-qPCR) was used to measure MAL2 expression experimentally. Gene set enrichment analysis (GSEA) was used to identify the biological pathways correlated with MAL2 expression in breast cancer, e.g., the relationship between the level of immune infiltration and MAL2 in breast cancer. Experimental and computational techniques showed that MAL2 was expressed at high levels in breast cancer tissues compared with the surrounding tissues. As a result, high MAL2 expression can be used as an independent biomarker for breast cancer. MAL2 expression level was also found to correlate with lower immune infiltrating levels in breast cancer.
